# Treatment of Midshaft Clavicular Fractures With Elastic Titanium Nails

**DOI:** 10.5812/traumamon.15623

**Published:** 2014-08-01

**Authors:** Hassan Keihan Shokouh, Mohammad Nasir Naderi, Mahsa Keihan Shokouh

**Affiliations:** 1 Department of Orthopedics, Faculty of Medicine, Hamadan University of Medical Sciences, Hamadan, IR Iran; 2 Department of Orthopedics, Kasra Hospital, Tehran, IR Iran; 3 Faculty of Pharmacy, Tehran University of Medical Sciences, Tehran, IR Iran

**Keywords:** Fracture Fixation, Intramedullary, Clavicle, Elastic nail

## Abstract

**Background:**

One of the modern techniques for the treatment of clavicle fracture (Fx) is elastic titanium intramedullary nailing. But, there are different opinions about this technique. We studied this technique in 12 patients with clavicle Fx and assessed its outcome.

**Objectives:**

We aimed to study the prognosis of midshaft clavicular Fx treated via minimally invasive stable elastic intramedullary nailing.

**Patients and Methods:**

We operated on 13 clavicle Fx in 12 patients from 2008 through 2012. We used a new technique called minimally invasive titanium elastic intramedullary nailing for operating patients with midshaft clavicular Fx.

**Results:**

Clinical union was achieved 3-5 weeks after the operation with no pain over Fx sites upon physical examination. Radiologic union appeared at 6 to 12 weeks .We did not encounter nonunion or infection, but one of the comminuted Fx united 1 cm shorter; however, it had a solid union with a good score. All but two patients had good scores.

**Conclusions:**

Although controversy exist regarding intramedullary nailing of clavicle Fx, our results using this technique for minimally comminuted midshaft clavicular Fx were very good.

## 1. Background

There are multiple techniques for the treatment of clavicle Fx (1-8); the trend is toward an operative approach (1, 3, 5, 8) which consists of two main procedures: plating or intramedullary nailing. Although plating is accepted as a standard technique, it has some disadvantages like large scar, nonunion, and difficult application and removal of the plate (1). The second method, intramedullary nailing of clavicular Fx, is done by many techniques and multiple devices (2, 6, 9-11) which have their own advantages (1, 6, 8, 10) and disadvantages (2, 10-12). Nowadays, rigid pins are not used due to their breakage and migration (10-12); a relatively new technique uses elastic titanium nails (13, 14).

This technique was attractive when first presented by Jubel et al. (13) but now there are varying opinions about it (6, 10). Some articles have recommended it as a technique with little complications, rapid union rate, easy insertion and removal, small scar and no breakage (6, 14). But, Campbell lists a wide range of complications ranging from 9-78% in various studies (15). However, others report opposite results such as high nonunion, the breakage of device, and lengthy operation time (4). We used this technique in 13 clavicular Fx and studied the outcome.

## 2. Objectives

Our aim in this study was to determine the outcomes of 13 midshaft clavicular Fx treated by minimally invasive intramedullary nailing with elastic titanium nails.

## 3. Patients and Methods

**Figure 1.**
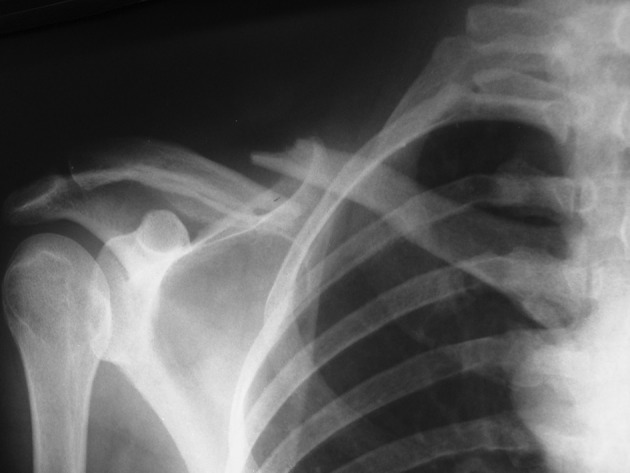
Comminuted Midshaft Clavicular Fracture

**Figure 2.**
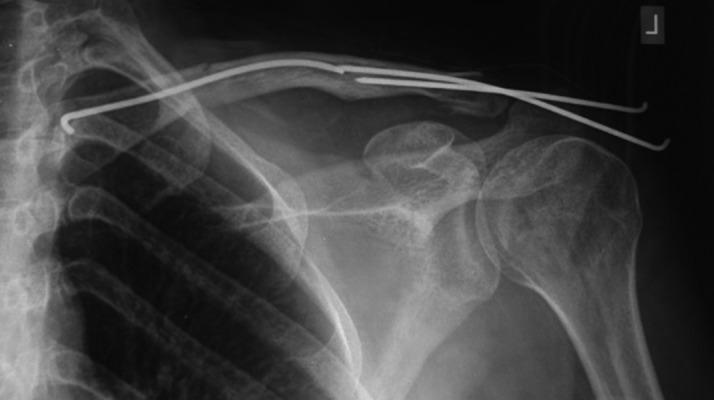
Postoperative Radiograph

**Figure 3.**
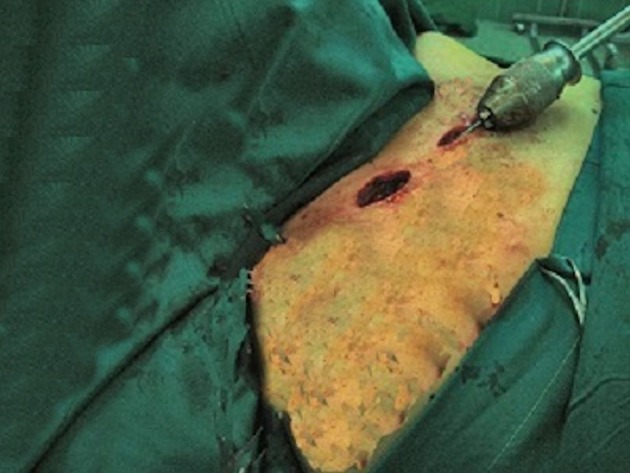
Preparation for Nail Insertion After Open Reduction of the Fracture

We operated 13 clavicular fractures in 12 patients from 2008 through 2012. Our exclusion criteria were old Fx, open Fx, proximal end and distal end Fx. The inclusion criteria were: closed, midshaft, acute clavicular Fx (Figure 1). Eight patients were women and 6 had comminuted Fx. The mean age of the patients was 29 years (range 17-42 years). Seven patients had high-energy trauma and three of them had multiple trauma. One patient had a bilateral Fx with right side ipsilateral acromioclavicular joint dislocation. The pattern of Fx in one patient was segmental, with ipsilateral midshaft and distal end clavicular fracture (Figure 2). Most cases were operated within 24-48 hours after trauma. In four patients, clavicle Fx was fixed by closed reduction and use of C-arm radiological control. In the remaining, open reduction was used for reduction and fixation. As a routine procedure, we used a small incision over the Fx site. The technique for this operation was as follows: A small incision was made over the skin 1 cm lateral to the medial end of the clavicle; then, for insertion of the nail, an entry point was made in the anterior cortex of the bone by a 3.2 mm drill. After preparation of the entry hole, a nail (2 to 3 mm in diameter) was advanced into the medullary canal of the medial segment of the clavicle and passed through the fracture site and lateral segment via oscillation using a universal chuck and T-handle (Figure 3).

**Figure 4.**
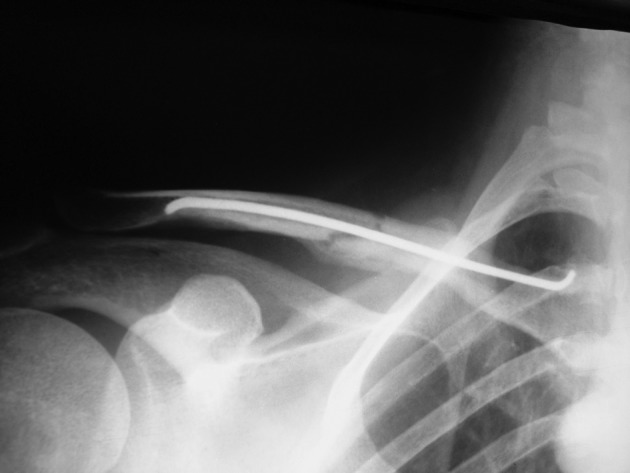
United Fracture

In most instances, another small skin incision was made at the Fx site to help fracture reduction. The elastic titanium nails with curved tips were passed into the clavicle. After reduction and fixation of the distal fragment, the nail was cut into the proper size and placed under the skin. During the postoperative period, patients were free to move their shoulders as much as they could. Immobility was not required, but over-head activity was restricted for 3-4 weeks. We followed the patients until union was achieved radiographically (Figure 4). The elastic nails were removed after three months.

## 4. Results

We used Constant Score to assess the clinical outcomes of our patients after union of the Fx (16). Clinical union was achieved in 3-5 weeks and radiographic union appeared in 6-12 weeks. One of the comminuted Fx united 1 cm short and its constant score was 90 with solid union. We had no infection or nonunion. All except two of our scores were excellent. Fractures of 4 patients with comminuted Fx united short (0.5 cm in 3 and 1 cm in one fracture) because of high-energy trauma. In 4 patients the length of scars was 1 cm over the entry point of the nail and in 9 patients an additional scar was present over the Fx site of open reduction. Two patients had long scars. Because one of them had simultaneous acromioclavicular joint dislocation and another had segmental fracture. Characteristics of patients are summarized in Table 1.

**Table 1.** Characteristics of the Patients With Intramedullary Nailing of Clavicle Midshaft Fractures With Elastic Titanium Nails ^a^

No.	Age, y	Sex	Side	Type of Trauma	Diagnosis	Duration of X-ray Union, wk	Pain (15)	Shortening, cm	Activities + Positioning (20)	ROM (40)	Power	Constant Score (100)
**1**	**29**	F	Lt	HE	Midshaft (simple)	12	15	0	20	36	24	95
**2**	**29**	F	Rt	HE	Midshaft FX (simple) + ACJ dx	8	14	0	18	36	22	90
**3**	**25**	F	Lt	HE	Midshaft FX (comminuted)	12	15	0	20	40	25	100
**4**	**28**	F	Lt	LE	Midshaft (simple)	8	15	0	20	40	25	100
**5**	**30**	F	Lt	HE	Midshaft + distal end FX (segmental)	10	15	0	20	40	25	100
**6**	**34**	F	Lt	HE	Midshaft (simple)	10	15	0	20	40	25	100
**7**	**17**	M	Lt	LE	Midshaft (simple)	6	15	0	20	40	25	100
**8**	**42**	F	Lt	HE	Midshaft FX (comminuted)	9	15	0.5	20	40	25	100
**9**	**36**	F	Rt	LE	Midshaft (simple)	7	15	0	20	40	25	100
**10**	**35**	F	Lt	HE	Midshaft FX (comminuted)	8	15	0	20	40	25	100
**11**	**35**	M	Rt	HE	Midshaft FX (comminuted)	9	15	0.5	20	40	25	100
**12**	**38**	M	Rt	HE	Midshaft FX (comminuted)	8	15	0.5	20	40	25	100
**13**	**34**	M	Rt	HE	Midshaft FX (comminuted)	10	12	1	16	38	24	90

^a^ Abbreviations: ACJ: acromioclavicular joint; dx, dislocation; F, female; FX, fracture; HE, high energy; LE, Low energy;LH, low energy; Lt, left; M, male; ROM, range of motion; Rt, right

## 5. Discussion

Clavicle Fx are not infrequent and account for approximately 2.6% of all Fx. The majority of clavicle fractures (80% to 85%) occur in the midshaft (17, 18). Clavicle fractures can be treated conservatively, but evidence regarding the superiority of operative treatment over conservative treatment is mounting. Duan and his colleagues evaluated the effect of plating vs. intramedullary pinning or conservative treatment for midshaft clavicular Fx (1). They concluded that there were no differences between plating and intramedullary pinning in therapeutic effects, but plating had a higher complication rate than pinning. Plating was also associated with improved functional results compared to conservative treatment.

 In a meta-analysis of the literature 2144 Fx in thirty years (1975-2005) , Zlowodzki and his colleagues showed that nonunion rate decreased from 15.2% to 2% by primary intramedullary nailing (8). In studying 31 midshaft clavicular Fx treated by intramedullary nailing with titanium elastic nail (TEN), Mueller et al. (6) concluded that intramedullary fixation of midshaft clavicle fracture with TEN was a safe and minimally invasive. This technique produced excellent cosmetic and functional results; thus, it could be an alternative to plate or screw fixation or nonsurgical treatment. 

However, some are against intramedullary nailing. Frigg et al. reported 34 patients treated with intramedullary nailing from April 2004 to March 2007 (2). They concluded that intramedullary nailing of midshaft clavicular fractures using the TEN had various complications postoperatively and was technically demanding. They also reported that in 70% of the patients, problems or complications occurred (seven medial perforations, seven laterals penetrations, one nail breakage, one nail dislocation, and hardware irritation in seven patients). 

Plating is the standard technique for operation of clavicle Fx when surgery is required, but fixation of clavicle Fx by elastic titanium nails is a new technique and can be used on some occasions. We had favorable results with this technique in cases with midshaft clavicular fracture. This technique is demanding and we do not recommend it in old comminuted clavicular fractures. Our study had some limitations namely the low number of patients.

## References

[1] Duan X, Zhong G, Cen S, Huang F, Xiang Z (2011). Plating versus intramedullary pin or conservative treatment for midshaft fracture of clavicle: a meta-analysis of randomized controlled trials.. J Shoulder Elbow Surg..

[2] Frigg A, Rillmann P, Perren T, Gerber M, Ryf C (2009). Intramedullary nailing of clavicular midshaft fractures with the titanium elastic nail: problems and complications.. Am J Sports Med..

[3] Hill JM, McGuire MH, Crosby LA (1997). Closed treatment of displaced middle-third fractures of the clavicle gives poor results.. J Bone Joint Surg Br..

[4] Khalil A (2009). Intramedullary screw fixation for midshaft fractures of the clavicle.. Int Orthop..

[5] McKee MD, Pedersen EM, Jones C, Stephen DJ, Kreder HJ, Schemitsch EH (2006). Deficits following nonoperative treatment of displaced midshaft clavicular fractures.. J Bone Joint Surg Am..

[6] Mueller M, Rangger C, Striepens N, Burger C (2008). Minimally invasive intramedullary nailing of midshaft clavicular fractures using titanium elastic nails.. J Trauma..

[7] Schuind F, Pay-Pay E, Andrianne Y, Donkerwolcke M, Rasquin C, Burny F (1988). External fixation of the clavicle for fracture or non-union in adults.. J Bone Joint Surg Am..

[8] Zlowodzki M, Zelle BA, Cole PA, Jeray K, McKee MD, Evidence-Based Orthopaedic Trauma Working G (2005). Treatment of acute midshaft clavicle fractures: systematic review of 2144 fractures: on behalf of the Evidence-Based Orthopaedic Trauma Working Group.. J Orthop Trauma..

[9] Grassi FA, Tajana MS, D'Angelo F (2001). Management of midclavicular fractures: comparison between nonoperative treatment and open intramedullary fixation in 80 patients.. J Trauma..

[10] Leppilahti J, Jalovaara P (1999). Migration of Kirschner wires following fixation of the clavicle--a report of 2 cases.. Acta Orthop Scand..

[11] Lyons FA, Rockwood CA, Jr. (1990). Migration of pins used in operations on the shoulder.. J Bone Joint Surg Am..

[12] Naidoo P (1991). Migration of a Kirschner Wire from the clavicle into the abdominal aorta.. Arch Emerg Med..

[13] Jubel A, Andermahr J, Schiffer G, Tsironis K, Rehm KE (2003). Elastic stable intramedullary nailing of midclavicular fractures with a titanium nail.. Clin Orthop Relat Res..

[14] Kadakia AP, Rambani R, Qamar F, McCoy S, Koch L, Venkateswaran B (2012). Titanium elastic stable intramedullary nailing of displaced midshaft clavicle fractures: A review of 38 cases.. Int J Shoulder Surg..

[15] Terry Canale S, James H (2013). Campbell, s Operative orthopedics..

[16] Constant CR, Murley AH (1987). A clinical method of functional assessment of the shoulder.. Clin Orthop Relat Res..

[17] Robinson CM (1998). Fractures of the clavicle in the adult. Epidemiology and classification.. J Bone Joint Surg Br..

[18] Craig EV, Rockwood CA, Matsen FA (1998). Fractures of the clavicle.. The Shoulder..

